# Fibromodulin reduces scar size and increases scar tensile strength in normal and excessive‐mechanical‐loading porcine cutaneous wounds

**DOI:** 10.1111/jcmm.13516

**Published:** 2018-02-01

**Authors:** Wenlu Jiang, Kang Ting, Soonchul Lee, Janette N. Zara, Richard Song, Chenshuang Li, Eric Chen, Xinli Zhang, Zhihe Zhao, Chia Soo, Zhong Zheng

**Affiliations:** ^1^ State Key Laboratory of Oral Diseases Department of Orthodontics West China Hospital of Stomatology Sichuan University Chengdu Sichuan China; ^2^ Division of Growth and Development Section of Orthodontics School of Dentistry University of California, Los Angeles Los Angeles CA USA; ^3^ Department of Orthopaedic Surgery CHA Bundang Medical Center CHA University Gyeonggi‐do South Korea; ^4^ Department of Bioengineering School of Engineering University of California, Los Angeles Los Angeles CA USA; ^5^ UCLA Division of Plastic and Reconstructive Surgery Department of Orthopaedic Surgery The Orthopaedic Hospital Research Center University of California, Los Angeles Los Angeles CA USA

**Keywords:** wound healing, tissue regeneration, scarring, hypertrophic scarring, fibromodulin

## Abstract

Hypertrophic scarring is a major postoperative complication which leads to severe disfigurement and dysfunction in patients and usually requires multiple surgical revisions due to its high recurrence rates. Excessive‐mechanical‐loading across wounds is an important initiator of hypertrophic scarring formation. In this study, we demonstrate that intradermal administration of a single extracellular matrix (ECM) molecule—fibromodulin (FMOD) protein—can significantly reduce scar size, increase tensile strength, and improve dermal collagen architecture organization in the normal and even excessive‐mechanical‐loading red Duroc pig wound models. Since pig skin is recognized by the Food and Drug Administration as the closest animal equivalent to human skin, and because red Duroc pigs show scarring that closely resembles human proliferative scarring and hypertrophic scarring, FMOD‐based technologies hold high translational potential and applicability to human patients suffering from scarring—especially hypertrophic scarring.

Hypertrophic scarring is a common postoperative complication, especially in non‐Caucasian patients, requiring multiple surgical revisions because of high recurrence rates 1, 2. Unfortunately, current available therapies are minimally effective for treating or preventing scarring or have undesirable side effects 3. To address the need for a safer, more effective treatment that can actively modulate the early wound environment to reduce scar formation, we explored potential solutions in developmental biology by studying foetal scar repair mechanisms in contrast to those of adult wound repair. The primary difference between adult and foetal skin wounds is that the former heals with scarring while the latter does not 4. In a completely new discovery, we found that an ECM molecule, FMOD, is critical to foetal‐type scarless repair 5. In particular, our previous studies revealed that FMOD has a unique mechanism of action to elicit a more ‘foetal‐like’ pro‐migratory and pro‐contractility phenotype in adult dermal fibroblasts 6. Essentially, FMOD reduces scar formation without diminishing the tensile strength in adult wound models such as mice, rats and even the Yorkshire pig, which has been shown to simulate normal human skin wound healing 6.

Pig models are required by the FDA for human skin product testing because among mammalian skins, porcine skin most closely approximates human skin in anatomic structure, mechanical properties and wound healing 7. However, previous studies have revealed significantly distinct dermal fibroblast behaviours and wound‐healing outcome between different pig wound models, specifically the Yorkshire and red Duroc pigs. For instance, in comparison with the dermal fibroblasts of Yorkshire pigs, the dermal fibroblasts of red Duroc pigs have greater expression of fibrosis‐related genes, higher converting rate to myofibroblasts and advanced contraction ability 8. In addition, Xie *et al*. reported that, like in human hypertrophic scars, the microvasculature is increased in the scar of adult female red Duroc pigs, but not in Yorkshire pig scars 9. Taken together, the red Duroc pig model most closely approximates the hypertrophic/fibroproliferative scarring seen in some human skin wounds while the Yorkshire pig model more closely resembles normal human scarring 7, 8, 9, 10. Therefore, in this study, adult female red Duroc pigs were chosen to further assess the efficacy of FMOD to further advance the translational potential and applicability of FMOD for hypertrophic scar reduction and prevention. All animal surgeries were performed in accordance with the NIH Guide for the Care and Use of Laboratory Animals set forth under the institutionally approved protocols provided by the Chancellor's Animal Research Committee at UCLA (protocol number: 2008‐016). Because accumulating data suggest that excessive‐mechanical‐loading across wounds is an important initiator of hypertrophic scarring formation 11, 12, 13, a large ellipse (2.0‐cm width × 1.5‐cm length) and a normal‐size ellipse (0.5‐cm width × 1.5‐cm length) were excised on the dorsal pig skin followed by primary closure (Fig. S1). This fourfold increase in wound width produced a 2.5‐fold increase in mechanical loading across the wound 13, creating a more challenging scenario for our experiment.

The Food and Drug Administration (FDA) has approved intralesional corticosteroid injections to repress scar formation, with triamcinolone acetonide (TAC) being the most commonly used. However, corticosteroid application has been shown to have significant side effects, including hypopigmentation, granulomas, ineffectiveness and skin atrophies 3, 14. Moreover, by prohibiting keratinocyte and fibroblast proliferation and collagen deposition, corticosteroids markedly suppress wound healing and impair scar tensile strength 3, 14, 15. Thus, determining whether corticosteroid administration improves hypertrophic scarring is a clinically relevant topic for wound‐healing management. In our experiment, we demonstrated that acute TAC injections did not considerably improve the gross appearance of excessive‐mechanical‐loading porcine wounds (Fig. 1A and B) as assessed by an adaption of the Visual Analogue Score 6 (VAS, Fig. S2), although TAC‐treated normal wounds exhibited lower VAS (Fig. S3A and B). Moreover, TAC failed to show efficacy in reducing scar size (Fig. S3A and C), when quantified by the Scar Index, which takes into account both scar area and dermal thickness measurement 6. Most importantly, TAC significantly reduced scar tensile strength of normal wounds by 65% (Fig. S3C and D) and in excessive‐mechanical‐loading wounds by 27% (Fig. 1C and D), 8 weeks post‐injury. Taking all this into consideration, acute corticosteroid usage likely has no significant advantages on wound‐healing management, including in excessive‐mechanical‐loading situations that tightly relate to hypertrophic scarring 11, 12, 13.

**Figure 1:**
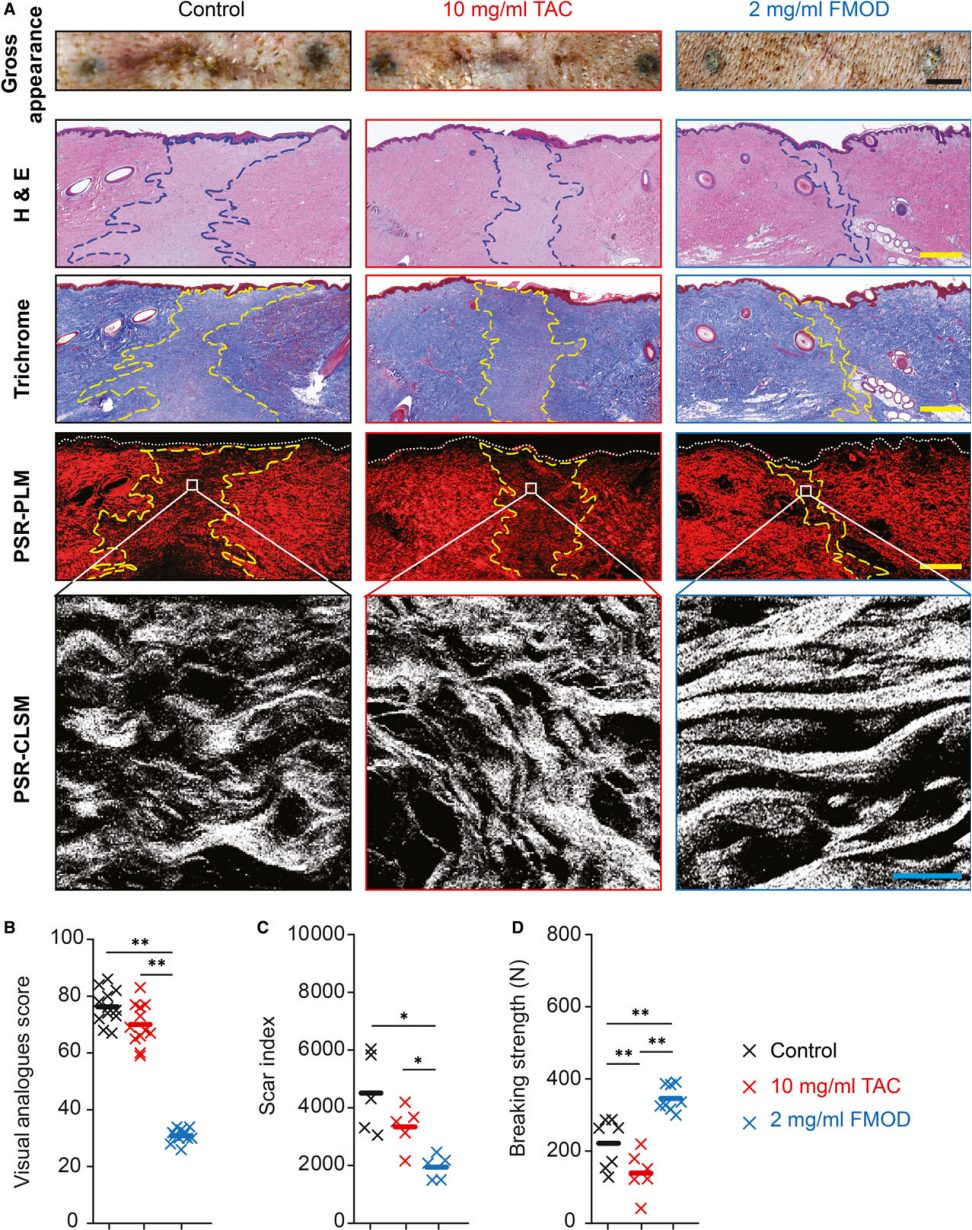
Efficacy of FMOD in reducing scar size and improving scar appearance in excessive‐mechanical‐loading adult female red Duroc porcine primary intention wounds at 8 weeks post‐injury. Gross visual appearance of wounds treated by PBS vehicle control, 2 mg/ml fibromodulin (FMOD) or 10 mg/ml triamcinolone acetonide (TAC) is shown, along with the corresponding histological evaluation by haematoxylin and eosin (H&E) staining, Masson's trichrome staining and picrosirius red (PSR) staining coupled with polarized light microscopy (PLM). Scar areas are outlined by dashed lines. PSR‐coupled confocal laser scanning microscopy (CLSM) was used to document upper dermal collagen architecture of wounds treated by PBS vehicle control, 2 mg/ml FMOD or 10 mg/ml TAC. (**A**). Gross visual appearance and scar size were quantified by the Visual Analogue Score (**B**) and Scar Index (**C**), respectively. Tensile strength was assessed as breaking strength (**D**). Wound areas are outlined. Scale bar = 25 mm (black), 0.5 mm (yellow) and 25 μm (cyan), respectively. In total, multiple wounds from 4 pigs were used for analysis. *P <* *0.05; **P < 0.005.

On the contrary, one‐time administration of FMOD at the surgical time markedly improved the visual scar appearance of normal red Duroc pig wounds, which were identical to wounds previously assessed in adult female Yorkshire pigs 6 8 weeks post‐injury (Fig. S3A and B). Similar improvements were seen in FMOD‐treated extensive‐mechanical‐loading wounds, as demonstrated by a 62% decrease in VAS compared with the phosphate‐buffered saline (PBS) vehicle control group (Fig. 1A and B). With regard to scar size, histological evaluation was performed by haematoxylin and eosin (H&E) staining, Masson's trichrome staining and picrosirius red (PSR) staining coupled with polarized light microscopy (PLM), and subsequently quantified by the Scar Index. We found that administration of FMOD consistently demonstrated significantly reduced scar size of normal red Duroc pig wounds 8 weeks post‐injury (Fig. S3A and C). Remarkably, the anti‐scarring effects of FMOD were even more pronounced in excessive‐mechanical‐loading wounds. Administration of FMOD resulted in 57% Scar Index reduction in excessive‐mechanical‐loading wounds (Fig. 1C), which was more than in normal wounds (44% Scar Index reduction). Meanwhile, confocal laser scanning microscopy (CLSM) demonstrated increased fractal dimension (*F*_*D*_) and decreased lacunarity (*L*) values in FMOD‐treated wounds compared to those of control groups (Fig. S4), indicating a finer texture in FMOD‐treated wounds 6. On the contrary, application of TAC only elevated *F*_*D*_ values without reducing *L* values (Fig. S4) suggesting a more complex, but spatially unorganized collagen architecture. As expected, the scar tensile strength increased in FMOD‐treated normal wounds by 27% (Fig. S3D) and increased in excessive‐mechanical‐loading wounds by 56% (Fig. 1D).

Collectively, our results demonstrated that, in comparison with vehicle control and traditional TAC injection, intradermal FMOD administration significantly reduced scar size, increased tensile strength, and improved dermal collagen architecture organization in the preclinical normal and excessive‐mechanical‐loading red Duroc porcine wounds. Since pig skin is recognized by the FDA as the closest animal equivalent to human skin 9, 10, 13, by presenting significant anti‐scarring bioactivity in both Yorkshire and red Duroc pig models, we believe FMOD‐based technologies will hold high translational potential for preventing and reducing human scar formation.

## Conflict of interest

KT, CS and ZZheng are inventors of fibromodulin‐related patents assigned to UCLA. KT, CS and ZZheng are founders and equity holders of Scarless Laboratories, Inc. which sublicenses fibromodulin‐related patents from the UC Regents. CS and ZZheng are also officers of Scarless Laboratories, Inc.

## Supporting information


**Figure S1** Scheme of primary intention wounds.

**Figure S2** Criteria used for Visual Analogue Scale (VAS) assessment in adult red Duroc pig primary intention wounds.

**Figure S3** Efficacy of FMOD in reducing scar size and improving scar appearance in normal adult female red Duroc porcine primary intention wounds at 8 weeks post‐injury.

**Figure S4** Fractural demotion (*F*_*D*_) and Lacunarity (*L*) analyses of adult female red Duroc porcine primary intension wounds at 8 weeks post‐injury.
