# Supplementary figures and images for: Fibromodulin reduces scar size and increases scar tensile strength in normal and excessive‐mechanical‐loading porcine cutaneous wounds
Source: J Cell Mol Med. 2018 Feb 1;22(4):2510–3. doi: 10.1111/jcmm.13516 (PMC5867110; doi:10.1111/jcmm.13516)

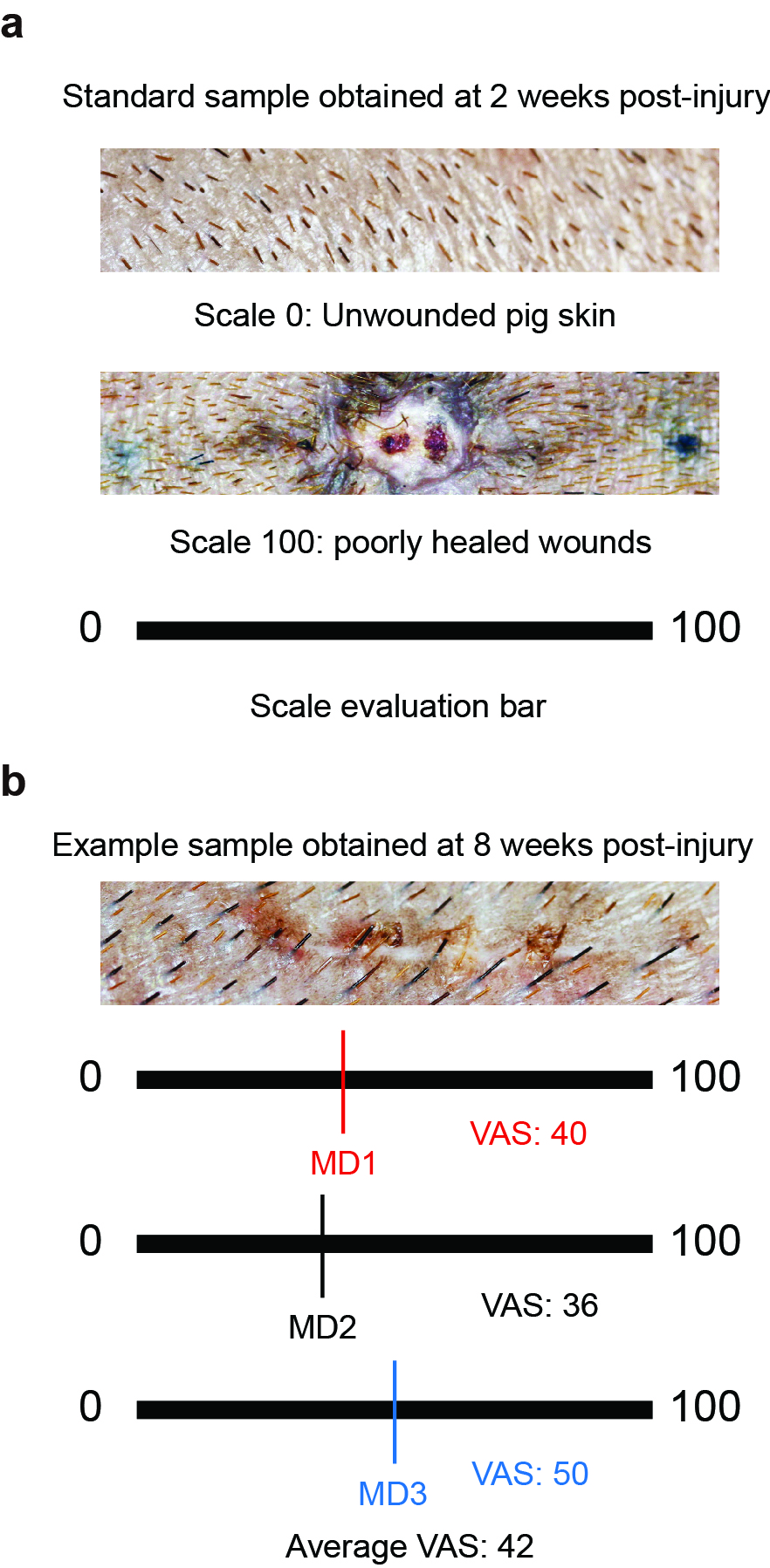

Supplement: Supplementary file 2 — Figure S2 Criteria used for Visual Analogue Scale (VAS) assessment in adult red Duroc pig primary intention wounds. [file JCMM-22-2510-s002.tif]

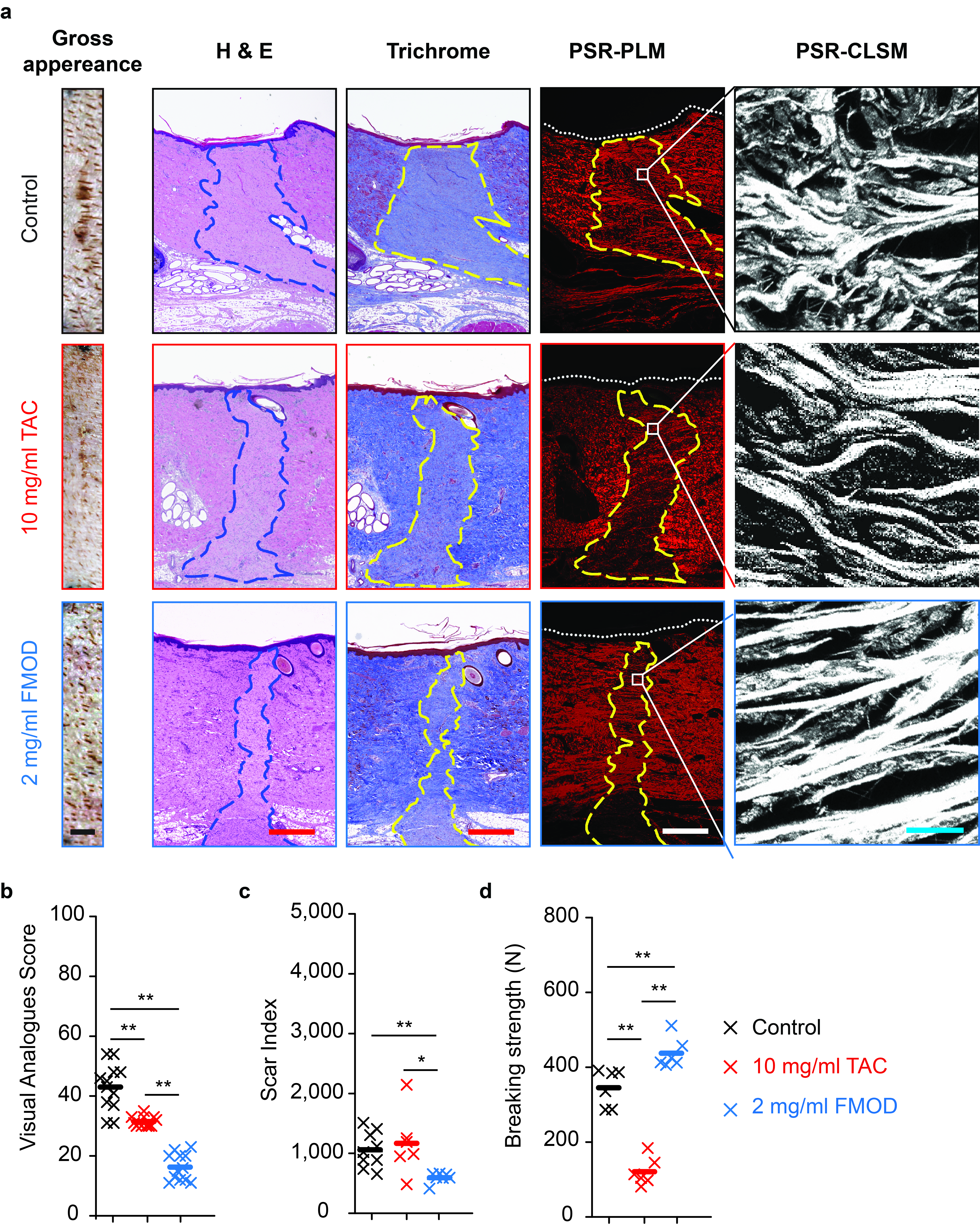

Supplement: Supplementary file 3 — Figure S3 Efficacy of FMOD in reducing scar size and improving scar appearance in normal adult female red Duroc porcine primary intention wounds at 8 weeks post‐injury. [file JCMM-22-2510-s003.tif]
