# Supplementary material for: Fibromodulin reduces scar size and increases scar tensile strength in normal and excessive‐mechanical‐loading porcine cutaneous wounds
Source: J Cell Mol Med. 2018 Feb 1;22(4):2510–3. doi: 10.1111/jcmm.13516 (PMC5867110; doi:10.1111/jcmm.13516)
Supplement: Supplementary file 5 [file JCMM-22-2510-s005.docx]

**Title:**

Fibromodulin reduces scar size and increases scar tensile strength in normal and excessive-mechanical-loading porcine cutaneous wounds

**Authors:**

Wenlu Jiang^1, 2, &^, Kang Ting^2, &^, Soonchul Lee^2, 3^, Janette N. Zara^4^, Richard Song^2^, Chenshuang Li^2^, Eric Chen^2^, Xinli Zhang^2^, Zhihe Zhao^1^, Chia Soo^5, *^, Zhong Zheng^2, *^

1. State Key Laboratory of Oral Diseases, West China Hospital of Stomatology, Sichuan University, Chengdu, Sichuan 610041, P. R. China.
2. Dental and Craniofacial Research Institute and Section of Orthodontics, School of Dentistry, University of California, Los Angeles, Los Angeles, CA 90095, USA
3. Department of Orthopaedic Surgery, CHA Bundang Medical Center, CHA University, Gyeonggi-do, 463-712, South Korea
4. Department of Bioengineering, School of Engineering, University of California, Los Angeles, Los Angeles, CA 90095, USA
5. UCLA Division of Plastic and Reconstructive Surgery and Department of Orthopaedic Surgery and the Orthopaedic Hospital Research Center, University of California, Los Angeles, Los Angeles, CA 90095, USA

**^&^ W. Jiang and K. Ting contributed equally to this work.**

***, Correspondence:**

Chia Soo, MRL 2641A, Box 951759, 675 Charles E Young Drive, South, Los Angeles, CA 90095-1759, USA. Phone: 1-310-7945479, Fax: 1-310-2067783, Email: [bsoo@ucla.edu](mailto:bsoo@ucla.edu).

Zhong Zheng, MRL 2641, 675 Charles E Young Drive, South, Los Angeles, CA 90095-1759, USA. Phone: 1-310-2065646, Fax: 1-310-2065349, Email: [zzheng@dentistry.ucla.edu](mailto:zzheng@dentistry.ucla.edu).

**Short Title**

Fibromodulin improves porcine wound healing

**Abbreviation used:**

CLSM Confocal Laser Scanning Microscopy

FDA Food and Drug Administration

FMOD Fibromodulin

*F_D_* Fractal dimension

H&E Hematoxylin and eosin

*L* Lacunarity

TAC Triamcinolone acetonide

PBS Phosphate buffered saline

PLM Polarized Light Microscopy

PSR Picrosirius red

VAS Visual Analogue Score

**Materials and Methods**

**Fibromodulin (FMOD) production**

cDNA of a human FMOD transcript (Genbank assessor number: NM_002023) was subcloned into a commercially available vector pSecTag2A (Life Technology, Grand Island, NY) with a C-terminal His-tag, and transfected into CHO-K1 cells (ATCC, Manassas, VA) [[1](#_ENREF_1)]. After a stable expression clone was established, the FMOD was produced and purified by a contract research organization, GenScript (Piscataway, NJ). Briefly, a stable human recombinant FMOD-expressing CHO-K1 cell line was cultured in a 1L serum-free Freestyle CHO Expression Medium (Thermo Fisher Scientific, Canoga Park, CA) at 37^o^C with 5% CO_2_ in an Erlenmeyer flask. The cell culture supernatant was then harvested on day 10 for purification with HiTrap^TM^ IMAC HP, 1-mL column (GE Healthcare, Uppsala, Sweden). The fractions from a 100 mM imidazole elution were collected and dialyzed against 20 mM phosphate-buffered saline (PBS), pH 7.4. Next, the sample with low conductivity was loaded onto HiTrap^TM^Q HP 1-mL column (GE Healthcare) for further purification. FMOD was then purified under non-reducing conditions, dialyzed again [[2](#_ENREF_2)], and then subjected to lyophilization. The purity of the FMOD product was 85%. Finally, FMOD was reconstituted in phosphate buffered saline (PBS), followed by sterilization through a 0.22-μm filter (Thermo Fisher Scientific) before usage.

**Animal surgery procedures**

All animal surgeries were performed under the institutionally approved protocols provided by the Chancellor’s Animal Research Committee at UCLA (Protocol number: 2008-016). Primary closure wound models were used in this study to simulate post-surgical wounds, which occur in 55 million elective operations and 25 million traumatic injury operations annually [[3](#_ENREF_3)]. Briefly, full-thickness wounds were created with a #15 surgical blade by excising a 0.5-cm width x 1.5-cm length or 2.0-cm width x 1.5-cm length ellipse of dorsal skin of 20-kg female, red Duroc pigs (Pork Power Farms), with the long axis of the cut running perpendicular and down to the fascia [[4](#_ENREF_4),[5](#_ENREF_5)]. All wounds were separated by at least 2 cm to minimize adjacent wound effects. A total of 24 wounds were created in each animal, and wounds from 8 pigs were randomly divided into different groups: immediately after excision, each open wound edge was injected with 100 μl PBS, 2.0 mg/ml FMOD, 10 mg/ml triamcinolone acetonide (TAC) in PBS (100 μl x 2 edges = 200 μl total/wound) or an irrelevant treatment under investigation (data not shown). Wounds were then marked with permanent dye and closed primarily with 3-0 Nylon mattress sutures (**Figure S1**). No additional injections were done post-surgery. Sutures were removed 2 weeks post-injury and wounds were then harvested 8 weeks post-injury.

**Scar visual appearance evaluation**

Gross visual assessment of the scar was performed in a randomized, double-blind fashion with an adaption of Visual Analogue Score (VAS) as described previously [[5](#_ENREF_5),[6](#_ENREF_6)]. Briefly, all scar images were taken by a highly sensitive digital camera (DSLR DS126181, Canon) and presented on a screen, each for a maximum of 20 seconds, to 3 experienced Doctor of Medicine (MD) assessors. The assessors placed a mark on a horizontal, 100-mm line to represent the scar quality, with 0 indicating unwounded skin and 100 indicating a poor scar (which represents a raised, hyperpigmented, or red scar with less acceptability and observer comfort; **Figure S2**).

**Tensile strength measurement**

Tensile strength was determined using an Instron 5565 Universal Testing Machine (Instron). An exact 4-cm length x 1-cm width full-thickness skin strip was obtained by meticulous dissection. Pneumatic compression grips were used to avoid slippage of the specimen. A 1-cm square area of skin was clamped on either side of the wound. The load to failure [breaking strength, as measured in Newtons (N)] was recorded [[5](#_ENREF_5)].

**Histological staining**

After fixation in 10% neutral-buffered formalin for 24 h, skin samples were dehydrated, paraffin-embedded, and sectioned into 5-μm increments for hematoxylin and eosin (H&E) and Masson’s trichrome staining, or into 10-μm increments for Picrosirius red (PSR) staining. To ensure a more precise quantitation, wounds were bisected centrally and the total scar area was normalized to varying dermal thickness using the previously described Scar Index [[1](#_ENREF_1),[5](#_ENREF_5),[7](#_ENREF_7)]. Images of light microscopy and polarized light microscopy (PLM) were collected by an Olympus BX51 microscope coupled with an Olympus DP73 camera and the cellSens Standard 1.9 software.

**Confocal laser scanning microscopy (CLSM)**

Following PSR staining, the collagen organization architecture of the upper dermis was documented by a Carl Zeiss LSM 510 META laser scanning confocal microscope and quantified with fractal dimension (*F_D_*) and lacunarity (*L*) analyses [[5](#_ENREF_5),[8](#_ENREF_8)], since these analyses are more sensitive than traditional methods, such as polarized light microscopy (PLM), X-ray diffraction, laser scattering, and the Fourier transform analysis [[7](#_ENREF_7)]. *F_D_* and *L* analyses were performed using the Frac_Lac add-on to ImageJ ([*http://rsbweb.nih.gov/ij/plugins/fraclac/fraclac.html*](http://rsbweb.nih.gov/ij/plugins/fraclac/fraclac.html)). For each sample, ten middle sections were chosen to capture dimensionality for the analysis. Colored images were first converted to gray scale and subsequently to binary images. Finally, collagen fibers were outlined and analyzed. A slow-scan, auto-threshold sliding-box method was used with a minimum box size of one pixel to maximum size of 45% of the region of interest [[5](#_ENREF_5),[8](#_ENREF_8)].

**Statistical analysis**

All statistical analyses were conducted in consultation with the UCLA Statistical Biomathematical Consulting Clinic and computed by the OriginPro 8. Data were generally presented as a mean and compared using the Mann-Whitney and Kruskal-Wallis ANOVA tests. *P* < 0.05 was considered statistical significance.

**References**

1. **Zheng Z, Nguyen C, Zhang X, Khorasani H, Wang JZ, Zara JN, Chu F, Yin W, Pang S, Le A, Ting K, Soo C.** Delayed wound closure in fibromodulin-deficient mice is associated with increased TGF-beta3 signaling. *J Invest Dermatol*. 2011; 131: 769-78.

2. **Li CS, Yang P, Ting K, Aghaloo T, Lee S, Zhang Y, Khalilinejad K, Murphy MC, Pan HC, Zhang X, Wu B, Zhou YH, Zhao Z, Zheng Z, Soo C.** Fibromodulin reprogrammed cells: A novel cell source for bone regeneration. *Biomaterials*. 2016; 83: 194-206.

3. **Sund B, Arrow AK.** New Developments in Wound Care: Clinica reports; 2000.

4. **Gurtner GC, Dauskardt RH, Wong VW, Bhatt KA, Wu K, Vial IN, Padois K, Korman JM, Longaker MT.** Improving cutaneous scar formation by controlling the mechanical environment: large animal and phase I studies. *- Ann Surg 1993 Apr;217(4):391-6*. 2011; 254: 217-25.

5. **Zheng Z, James AW, Li C, Jiang W, Wang JZ, Chang GX, Lee KS, Chen F, Berthiaume EA, Chen Y, Pan HC, Chen EC, Li W, Zhao Z, Zhang X, Ting K, Soo C.** Fibromodulin reduces scar formation in adult cutaneous wounds by eliciting a fetal-like phenotype. *Signal Transduction and Targeted Therapy* 2017; 2: e17050.

6. **Duncan JA, Bond JS, Mason T, Ludlow A, Cridland P, O'Kane S, Ferguson MW.** Visual analogue scale scoring and ranking: a suitable and sensitive method for assessing scar quality? *Plast Reconstr Surg*. 2006; 118: 909-18.

7. **Khorasani H, Zheng Z, Nguyen C, Zara J, Zhang X, Wang J, Ting K, Soo C.** A quantitative approach to scar analysis. *Am J Pathol*. 2011; 178: 621-8.

8. **Zheng Z, Zhang X, Dang C, Beanes S, Chang GX, Chen Y, Li C-S, Lee KS, Ting K, Soo C.** Fibromodulin is essential for fetal-type scarless cutanous wound healing. *American Journal of Pathology*. 2016; 186: 2824-32.

9. **Smith TG, Jr., Lange GD, Marks WB.** Fractal methods and results in cellular morphology--dimensions, lacunarity and multifractals. *Journal of neuroscience methods*. 1996; 69: 123-36.

10. **Ling EJY, Servio P, Kietzig AM.** Fractal and Lacunarity Analyses: Quantitative Characterization of Hierarchical Surface Topographies. *Microsc Microanal*. 2016; 22: 168-77.

**Supplementary Figure Legends**

**Figure S1. Scheme of primary intention wounds.**

For a normal wound, 0.5-cm width x 1.5-cm length ellipse was excised on adult female red Duroc pig dorsal skin down to the fascia (**a**). For a high-mechanical-loading wound, the wound width was extended to 2.0 cm (**b**). 100 μl of vehicle control buffer, 2.0 mg/ml FMOD, or 10 mg/ml TAC was intradermally injected into each wound edge at time 0 (200 μl/wound) before primary closure.

**Figure S2.** **Criteria used for Visual Analogue Scale (VAS) assessment in adult red Duroc pig primary intention wounds.**

On a standard sample obtained from adult female red Duroc pig at 2 weeks post-injury, a value between 0 (unwounded pig skin) and 100 (poorly healed wounds) was given, with lower values indicating subjective improvements in scar appearance and reduction in scar size (**a**). Three experienced Doctor of Medicine (MD) assessors scored each scar in a randomized, double-blind fashion, and the scores were averaged to generate a final VAS score as shown in this example sample obtained at 8 weeks post-injury (**b**).

**Figure S3. Efficacy of FMOD in reducing scar size and improving scar appearance in normal adult female red Duroc porcine primary intention wounds at 8 weeks post-injury.**

Gross visual appearance of wounds treated by PBS vehicle control, 2 mg/ml fibromodulin (FMOD), or 10 mg/ml triamcinolone acetonide (TAC) are shown, along with the corresponding histological evaluation by hematoxylin and eosin (H&E) staining, Masson’s trichrome staining and picrosirus red (PSR) staining coupled with polarized light microscopy (PLM). Scar areas are outlined by dashed lines. PSR-coupled confocal laser scanning microscopy (CLSM) was used to document upper dermal collagen architecture of wounds treated by PBS vehicle control, 2 mg/ml FMOD, or 10 mg/ml TAC. (**a**). Gross visual appearance and scar size were quantified by the Visual Analogue Score (**b**) and Scar Index (**c**), respectively. Tensile strength was assessed as breaking strength (**d**). Wound areas are outlined. Scale bar = 25 mm (black), 0.5 mm (red and white), and 25 μm (cyan), respectively. In total, multiple wounds from 4 pigs were used for analysis. *, *P* < 0.05; **, *P <* 0.005.

**Figure S4. Fractural demotion (*F_D_*) and Lacunarity (*L*) analyses of adult female red Duroc porcine primary intension wounds at 8 weeks post-injury.**

*F_D_* (**a**) and *L* (**b**) analyses were performed using PSR-CLSM images of normal (1.5-cm length x 0.5-cm width) and high-mechanical-loading (1.5-cm length x 2.0-cm width) adult red Duroc porcine wounds. *F_D_* provides a measure of how completely an object fills space, which quantifies an object in terms of shape, regularity, lack of smoothness, size, and number of self-similarities, *i.e.* invariance regardless of scale [[9](#_ENREF_9)]. In contrast, *L* permits an analysis of density, packing or dispersion through scales. In other words, *L* is a measure of the heterogeneity of a structure or the degree of structural variance within an object, which is related to the distribution of empty spaces (lacunas) of an image [[9](#_ENREF_9)]. Objects with lower *L* values correspond to a finer texture while higher *L* values are more spatially unorganized [[10](#_ENREF_10)]. In total, multiple wounds from 4 pigs were used for analysis, **, *P <* 0.005.
